# One-step grafting of temperature-and pH-sensitive (N-vinylcaprolactam-co-4-vinylpyridine) onto silicone rubber for drug delivery

**DOI:** 10.1080/15685551.2016.1231033

**Published:** 2016-09-20

**Authors:** Victor H. Pino-Ramos, Carmen Alvarez-Lorenzo, Angel Concheiro, Emilio Bucio

**Affiliations:** ^a^ Departamento de Química de Radiaciones y Radioquímica, Instituto de Ciencias Nucleares, Universidad Nacional Autónoma de México, Circuito Exterior, Ciudad Universitaria, México DF 04510, Mexico; ^b^ Facultad de Farmacia, Departamento de Farmacia y Tecnología Farmacéutica, R+D Pharma Group (GI-1645), Universidade de Santiago de Compostela, Santiago de Compostela, Spain

**Keywords:** Dually responsive, silicone rubber, drug-eluting device, diclofenac sodium, cytocompatibility, bacteria adhesion

## Abstract

A one-step method was implemented to graft N-vinylcaprolactam (NVCL) and 4-vinylpyridine (4VP) onto silicone rubber (SR) films using gamma radiation in order to endow the silicone surface with temperature- and pH-responsiveness, and give it the ability to host and release diclofenac in a controlled manner and thus prevent bacterial adhesion. The effects of radiation conditions (e.g., dose and monomers concentration) on the grafting percentage were evaluated, and the modified films were characterized by means of FTIR-ATR, Raman spectroscopy, calorimetry techniques (DSC and TGA) and contact angle measurements. The films responsiveness to stimuli was evaluated by recording the swelling degree of pristine and modified SR in buffer solutions (critical pH point) and as a function of changes in temperature (Upper Critical Solution Temperature, UCST). The graft copolymers of SR-g-(NVCL-co-4VP) showed good cytocompatibility against fibroblast cells for prolonged times, could host diclofenac and release it in a sustained manner for up to 24 h, and exhibited bacteriostatic activity when challenged against *Escherichia coli*.

## 1. Introduction

The combination of medical devices with drugs, namely the preparation of drug-eluting medical devices, is receiving a great deal of attention as it can provide relevant therapeutic synergisms.[1,2] Implantable medical devices mainly exert the therapeutic function through a physical mechanism of support or replacement of an affected tissue. However, they are quite prone to the adhesion of proteins and cells from the host, which can lead to adverse foreing-body reactions (inflammation, restenosis, opacification, etc.), as well as to surface colonization by microorganisms, which in turn transforms the device on a focus of infection.[3] The incorporation of antiinflammatory, antimicrobial or antiproliferative drugs on a medical device may atenuate the host response and the microbial adhesion. In addition, the device can act as a suitable platform for the release of a variety of other drugs for a prolonged time in a specific site, enhancing the therapeutic performance compared to a systemic administration*.*[4,5] Consequently, the efficacy and the safety of the treatment, as well as its cost-effectiveness, are improved.

The first drug-combined medical devices were drug-eluting stents, which were approved in 2003, and since then the field has opened up to include various other medical devices, such as sutures and catheters.[6,7] Nonetheless, some of the challenges that hinder the further development of drug-eluting medical devices include the low affinity of most biomaterials for drugs, which limits drug adsorption on the surface, and the deleterious effects that incorporating high amounts of drug in the bulk material might cause on the performance of the medical device. Several strategies, among them polymer grafting, are under development to overcome these limitations.[8] Polymer nanobrushes grafted on the surface of the medical device, as either individual or cross-linked chains, serve as a suitable wrapping nanolayer that can host and control the release of drugs. The grafting can be inititated by means of chemicals or applying ionizing energy.[9] In this regard, gamma radiation is particularly attractive for the functionalization of medical devices [10,11] because it has the advantage of being widely used for a variety of high scale-up processes including sterilization, and does not require chemical adyuvants such as catalysts, thus avoiding subsequent steps for their removal.[6]

Drug release can be controlled by diffusion and/or competitive mechanisms or by the responsiveness of polymer brushes (stimuli-responsive polymers) to certain physiological or external stimuli.[12] Stimuli-sensitive polymers undergo abrupt conformational changes in response to tiny variations in pH, temperature, medium composition, light, and magnetic or electrical fields.[13,14] A variety of pH- and temperature-sensitive polymers have been already grafted onto solid surfaces; drug molecules can be readily loaded when the network is swollen, while drug diffusion occurs slowly when the network is collapsed at physiological conditions.[15]

The aim of this work was to implement a one-step method to graft N-vinylcaprolactam (NVCL) and 4-vinylpyridine (4VP) onto silicone rubber (SR), using gamma irradiation, in order to endow the silicone surface with temperature- and pH-responsive brushes that can host and release diclofenac in a controlled manner. SR is one of the most common components of insertable medical devices. Diclofenac is one of the most used non-steroidal anti-inflammatory drugs (NSAID), but it also exhibits bacteriostatic [16] and antimicrobial activity against many microorganisms.[17] Therefore, diclofenac-eluting medical devices may simultaneously exhibit reduced inflammatory response and lower risk of microbial colonization. NVCL and 4VP have not been grafted together before, and thus a first aim of the work was to elucidate the effects of the monomers concentration and absorbed dose on the grafting percentage. These two compounds were chosen because (i) NVCL is a monomer with amphiphilic character and in its polymer form is biocompatible and exhibits temperature-responsiveness, making it a safer alternative than PNIPAAm [18–23]; and (ii) poly(4-vinylpyridine) (P4VP) is a cationic polymer that exhibits pH-responsiveness.[24] Dual temperature- and pH-responsive copolymers have been extensively studied because of the shifts that the pH-responsive component produces in the temperature-responsive polymer, which could move the phase volume transition temperature in or out of the physiological range.[23,25] Hence, a second aim of the work was to elucidate the effect that co-grafting NVCL and P4VP might have on the original responsiveness of each component. Finally, since both NVCL and P4VP bear chemical groups that can interact with sodium diclofenac, the effect of grafting percentage on drug loading/release profiles was investigated, as well as the prevention of bacteria adhesion. Cytocompatibility tests were carried out to verify compatibility of the modified SR with mammalian cells.

## 2. Materials and methods

### 2.1. Materials

Silicone rubber films (SR, 1 mm in thickness) were from GoodFellow (Huntingdon, UK). N-vinylcaprolactam and 4-vinylpyridine were from Sigma–Aldrich Co. (St. Louis, MO, USA) and purified by vacuum distillation before use. Toluene (analytical grade) was from J.T. Baker Co. (Mexico) and used as received. Diclofenac sodium from Vorquímica, S.L. (Spain). The MTT proliferation kit was from Roche (Germany) and the fibroblast cells (BALB/3T3; ATCC CCl-163^TM^) were from the American Type Culture Collection (Manassas, VA, USA).

### 2.2. Synthesis of graft copolymer

Films of SR were washed with ethanol during 3 h and dried, weighed and placed in glass ampoules containing a mixture of NVCL and 4VP (1/1 v/v) in toluene (total monomers concentration ranged from 10 to 70% v/v). Then the ampoules were degassed by freeze-thaw cycles (5 times), sealed, and irradiated with a ^60^Co γ-source (Gammabeam 651 PT, MDS Nordion, Canada) at 10.3 kGy/h of intensity. The obtained SR-g-(NVCL-co-4VP) films were washed in ethanol for 12 h to remove residual monomers and homopolymer formed during the reaction, and then dried under vacuum to constant weight. The grafting percentage was calculated as follows:

(1) $$G\left(\%\right)=100[\left(W_{\text{g}}-W_{0}\right)/W_{0}]$$

where *W*_0_ and *W*_g_ represent the weights of the initial and grafted films, respectively.

### 2.3. Structural and thermal characterization

Infrared (FTIR) spectra in attenuated total reflection mode (ATR) of copolymers were recorded using a Perkin–Elmer Spectrum 100 spectrometer (Perkin Elmer Cetus Instruments, Norwalk, CT) with 16 scans. Raman spectra were recorded using a Renishaw inVia confocal Raman microscope (Renishaw Ibérica S.A.U., Spain). Thermal decomposition was determined in nitrogen atmosphere between 25 and 800 °C at a heating rate of 10 °C/min using a TGA Q50 (TA Instruments, New Castle, DE, USA). Differential scanning calorimetry (DSC) studies for the determination of thermal transitions were carried out under nitrogen flow using a DSC 2010 calorimeter (TA Instruments, New Castle, DE, USA) from 25 to 250 °C at a heating rate of 10 °C/min.

### 2.4. Degree of swelling and contact angle measurements

Pieces of SR and SR-g-(NVCL-co-4VP) copolymers were weighed and immersed in distilled water at 25 °C. At prestablished time intervals (from 15 min to 48 h), the samples were removed from the medium and weighed after removal of excess water with a piece of paper. The swelling percentage was calculated as follows:

(2) $$\text{Swelling}\;\left(\%\right)=[\left(W_{\text{s}}-W_{\text{d}}\right)/{\text{W}}_{\text{d}}]\cdot 100$$

where *W*_s_ and *W*_d_ are the weighs of the swollen and dried pieces, respectively.

The temperature responsiveness of SR-g-(NVCL-co-4VP) was estimated from the changes in swelling in phosphate buffer solution (PBS pH 7.4) as a function of temperature [26] in the 22–36 °C range. The upper critical solution temperature (UCST) value was considered to be the inflection point of the degree of swelling vs. temperature plot.

The critical pH value was determined from the changes in the swelling degree of copolymer films that were placed in buffer media of pH ranging from 4 to 10 at 25 °C, during 24 h. The buffers were prepared by mixing adequate volumes of boric acid (0.2 M) and citric acid (0.05 M) solutions with a trisodium phosphate dodecahydrated (0.1 M) solution.[27] The critical pH value was defined as the inflection point in the swelling degree vs. solution pH plot.

A DSA 100 (Krüss GmbH, Hamburg, Germany) was used to measure the water contact angle of SR-g-(NVCL-co-4VP) by depositing a droplet of distilled water onto the modified film (previously washed and dried) and measuring the water-surface contact angle immediately after. The measurement was done 3 times at different zones of the film and at 25 °C. Films with different graft percentages were analyzed.

### 2.5. Cytocompatibility assay

The assay was carried out using embryonic mouse fibroblast BALB/3T3 cells. First, SR and SR-g-(NVCL-co-4VP) copolymer pieces were washed with water under constant stirring for 24 h to remove impurities, and subsequently dried in an oven at 40 °C to constant weight. The samples were exposed to UV radiation during 20 min,[28] and immediately after placed in 300 μL of culture medium (DMEM F12 supplied with 10% FBS and 1% of antibiotic solution) and incubated at 37 °C for 24 h. BALB/3T3 cells in DMEM F12 Ham supplemented with 10% FBS and 1% antibiotic solution (200,000 cell/mL; 100 μL) were seeded in 96-well plates. After 24 h of incubation, 100 μL of medium in contact with the copolymers were poured into the wells containing cells, which were then incubated (37 °C, 5% CO_2_, 90% RH) during 24 or 48 h more. Afterwards, the culture medium was replaced with 100 μL of fresh medium, and 10 μL of the reagent 1 of the MTT kit was added. After 4 h of incubation at 37 °C, 100 μL of the reagent 2 of the MTT kit was added and incubated again at 37 °C during 4 h. Plates were read at 550 nm using an ELISA plate reader (BIORAD Model 680 Microplate Reader, USA). Tests were done in triplicate.[29] Negative controls were also prepared by just adding fresh culture medium to cells and treating them in the same way. Cell viability was calculated as follows:

(3) $$\text{Cell Viability}\;\left(\%\right)=\left({\text{Abs}}_{\text{sample}}/{\text{Abs}}_{\text{negative control}}\right)\cdot 100$$

### 2.6. Diclofenac loading and release

The graft copolymers were extensively washed with water, dried in an oven at 40 °C and finally weighed. Pieces of the films (0.3 cm^2^) were placed in vials with a diclofenac sodium aqueous solution (80 mg/L; 5 mL) and kept under constant stirring for 48 h, protected from light and at room temperature. At pre-established time intervals, samples were taken from the release medium and the absorbance was measured at 276 nm (UV-Vis spectrophotometer, Agilent 8453, Germany). The experiment was carried out in triplicate. Using a validated calibration curve, the amount of drug loaded was calculated as follows.

(4) $$\text{Diclofenac \textbackslash{}, loaded}\;\left(\text{mg}/\text{g}\right)=\left(C_{1}-C_{2}\right)\cdot V/W$$

where *C*_1_ and *C*_2_ represent the initial and final concentration of drug in the loading medium, respectively, *V* the volume of solution used for loading, and *W* the weight of film.

Diclofenac-loaded films were dried and then transferred to vials with saline serum (0.9% NaCl; 5 mL) and kept at 37 °C under constant stirring (300 rpm) and protected from light.[30,31] The amount of diclofenac released was monitored by measuring the absorbance of samples that were taken at different times from the release medium (276 nm; UV-Vis spectrophotometer, Agilent 8453, Germany). The samples were returned to the medium. The tests were carried out in triplicate.

### 2.7. Bacterial adhesion assay

Copolymer pieces (0.3 cm^2^) were placed in vials containing 5 mL of a diclofenac solution (0.04 mg/mL), which were then sealed, autoclaved at 120 °C for 20 min, and gently shaken for 48 h at room temperature. Then, the copolymer pieces were placed in vials with 2 mL of an *Escherichia coli* culture solution in trypticase soy broth (TSB; 8 × 10^8^ CFU/mL) and incubated at 37 °C for 3 h. Afterwards, the films were removed from the culture medium, washed with phosphate buffer solution (PBS), placed in vials with 2 mL of PBS and sonicated with a Bronson Sonifier 250 for 5 min to release the bacteria that had adhered to the films. Solution samples obtained from the sonication process were taken, and dilutions were made and seeded in Petri dishes with agar medium.[32] CFU counting was performed after incubation for 24 h at 37 °C.

### 2.8. Statistical analysis

Effects of grafting percentage on cytocompatibility, diclofenac loading and bacteria adhesion were analyzed using ANOVA and multiple range test (Statgraphics Centurion XVI 1.15, StatPoint Technologies Inc., Warrenton VA).

## 3. Results and discussion

### 3.1. Grafting process

**Figure 1.**
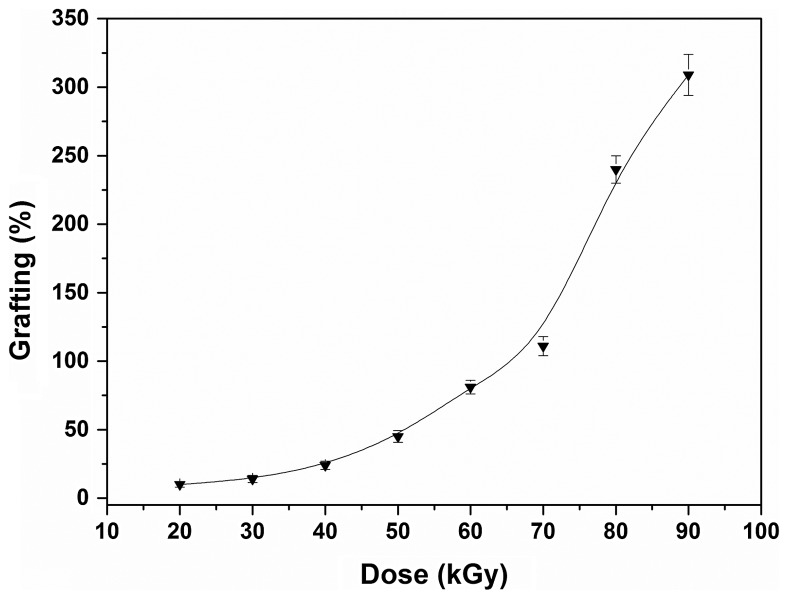
Grafting percentage of NVCL and 4VP onto silicone as a function of dose, NVCL/4VP monomers/toluene 7/3 (v/v), I = 10.3 kGy/h.

**Figure 2.**
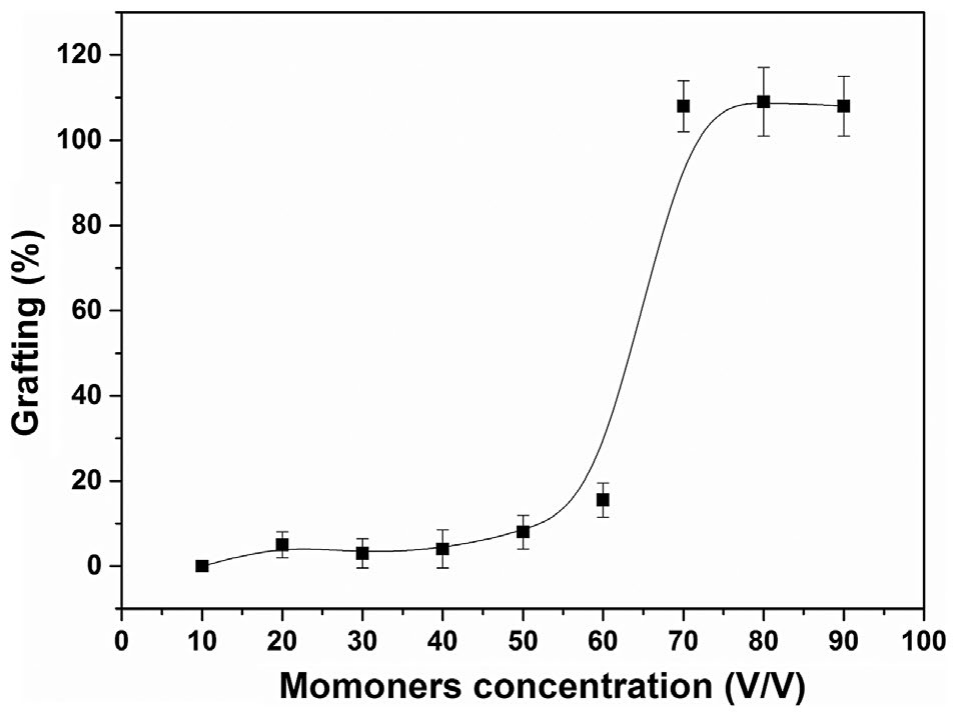
Grafting percentage of SR-g-(NVCL–co-4VP) as a function of monomers concentration in toluene (v/v), dose 70 kGy, I = 10.3 kGy/h.

SR-g-(NVCL-co-4VP) copolymers were synthesised applying a one-step radiation grafting method. To the best of our knowledge, NVCL and 4VP have not been grafted together before and, therefore, attention was paid to the effects of monomers concentration and absorbed dose on the graft percent. All experiments were carried out using a fixed NVCL:4VP 1:1 v/v ratio, but the monomers mixture was diluted with toluene at various proportions. The effect of the dose needed to carry out the polymerization reaction was evaluated by varying the dose rate using a constant toluene solution of 70% monomers (Figure 1). The grafting percentage steadily increased as the absorbed dose increased up to 70 kGy (≈100% grafting), but then a jump occurred and at 80 kGy the grafting percentage was above 200%. Further increases in dose led to additional increments in the amount of copolymer grafted. At high doses more C–H bonds are broken and higher number of reactive sites is created on the SR, meaning that more N-vinylcaprolactam and 4-vinylpyridine monomers can react. A similar dependence was observed when the effect of the monomer concentration was evaluated (Figure 2). For a fixed absorbed dose of 70 kGy, there was a rapid increase in the grafting percentage when the monomers concentration was raised from 60 to 70%. Similar dependences have been previously observed for other monomer combinations and are related to an increase in the probability of interaction of the monomer molecules with the radicals formed on the SR, starting thereby the grafting reaction. Thus, by tuning both the absorbed dose and monomers concentration a variety of grafting percentages can be obtained.

### 3.2. Characterization of SR-g-(NVCL-co-4VP) copolymers

**Figure 3.**
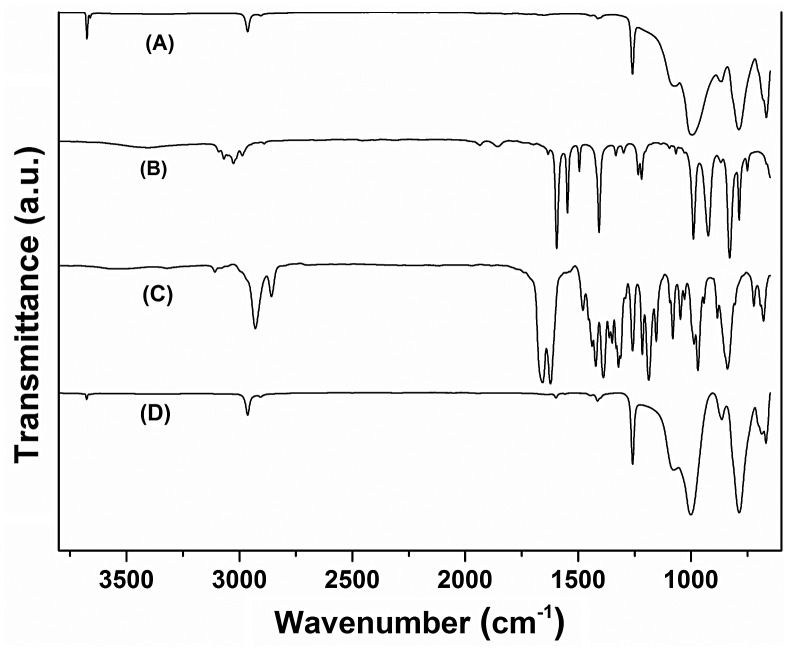
IR spectra of (A) pristine silicone rubber, (B) 4VP, (C) NVCL and (D) SR-g-(NVCL-co-4VP), (42%).

SR and SR-g-(NVCL-co-4VP) copolymers showed similar FTIR-ATR spectra probably because an overlapping of the bands of the grafted monomers with those of SR (Figure 3). Bands at 2963 and 1258 cm^−1^ corresponded to Si–C asymmetrical stretching of C–H bonds, and at 1008 cm^−1^ to the stretching vibration of the Si–O bond of silicon main chain. The absorption band at 1657 cm^−1^, which is typical of a C=C double bond stretching, did not appear in the graft copolymer, and the carbonyl group of the amide at 1622 cm^−1^ was not perceptible. Bands at 2908 cm^−1^ corresponded to aromatic C–H vibrations, while at 1598 and 1415 cm^−1^ corresponded to the stretching of C=N due to the pyridine ring grafted onto silicone. The presence of these bands confirmed the grafting by comparison with absorption bands of the raw materials (Figure 3).

**Figure 4.**
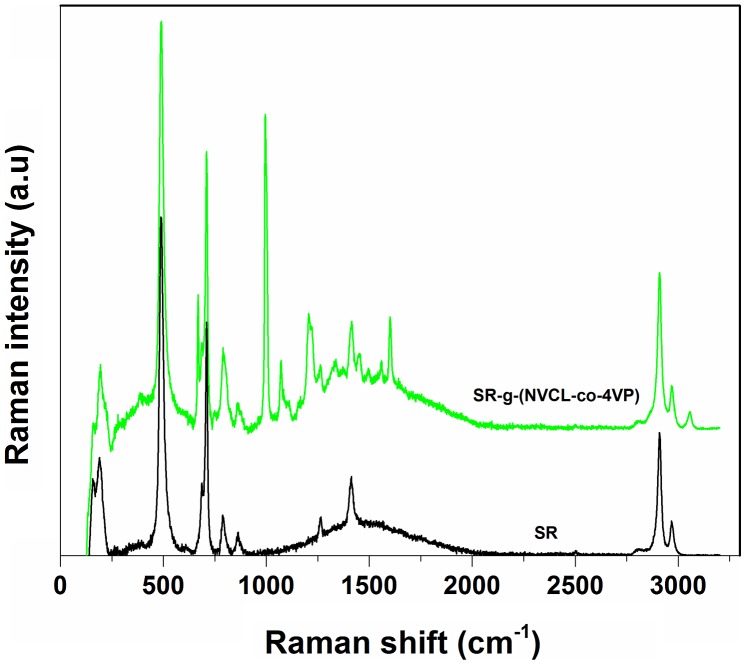
Raman spectra of pristine silicone rubber and SR-g-(NVCL-co-4VP) (42%) using a laser of neon at 785 nm.

A more detailed analysis was carried out by means of Raman spectroscopy of the film surface (Figure 4). The copolymers exhibited a new band at 3090 cm^−1^ that corresponded to C–H stretching from the aromatic ring of 4VP, bands at 2964 and 2905 cm^−1^ which corresponded to the symmetrical and asymmetrical stretching of C–H, at 1657 cm^−1^ due to the stretching band of C=O from NVCL grafted, at 1240 cm^−1^ associated to C–N stretching, at 1032 cm^−1^ due to aromatic C–H bending, at 710 cm^−1^ associated to stretching of Si–C and at 494 cm^−1^ because of the stretching of the Si–O bond.[33] Differences with respect to pristine SR confirmed the binary graft (Figure 4).

**Figure 5.**
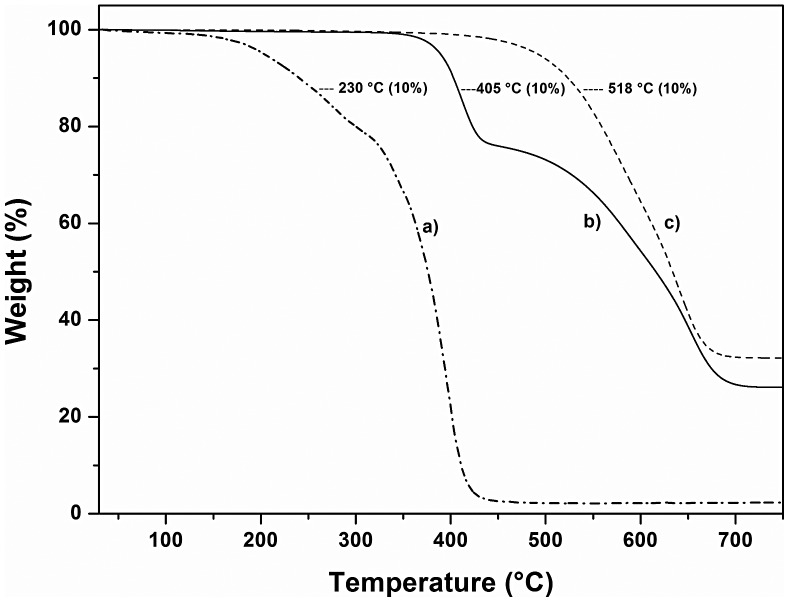
Thermogravimetric curves of (a) poly(NVCL-co-4VP), (b) SR-g-(NVCL-co-4VP) 42% grafting, and (c) SR pristine. The values in parentheses correspond to the weight loss percentage up to the given temperature.

The thermal stability of the materials was evaluated by means of TGA by recording the weight-loss as a function of temperature. The temperature at which 10 wt% weight loss occurred was 518 °C for SR, 405 °C for SR-g-(NVCL-co-4VP) with 42% graft, 382 °C for SR-g-(NVCL-co-4VP) with 300% graft, and 230 °C for a poly(NVCL-co-4VP) prepared under similar conditions (Figure 5). These findings indicate that the grafting has moderate detrimental effect on thermal stability due to the lower decomposition temperature of poly(NVCL-co-4VP) compared to that of SR. Nevertheless, the obtained copolymers may exhibit sufficient stability under common manufacturing processes for biomedical applications. The glass transition temperature (*T*_g_) was recorded using a DSC equipment. Typical *T*_g_ values of SR are between −80 and −85 °C,[34,35] which falls out of the range of the DSC apparatus used. The *T*_g_ value of SR-g-(NVCL-co-4VP) films was of 150 °C for a 108% graft, while for poly(NVCL-co-4VP) the *T*_g_ was 138 °C. Overall, the *T*_g_ values were similar for all graft percentages.

### 3.3. Water contact angle and swelling degree

Water contact angle of pristine SR was 108.3°, as expected from its hydrophobic character. Increasing the content in grafted NVCL-co-4VP copolymer caused a progressive decrease in the water contact angle; for 20, 45, 90, 240 and 340% grafting the water contact angle were 105.2°, 101.7°, 97.3°, 92.5° and 89.7º. Thus, the SR-g-(NVCL-co-4VP) films had a more hydrophilic surface than SR.

**Figure 6.**
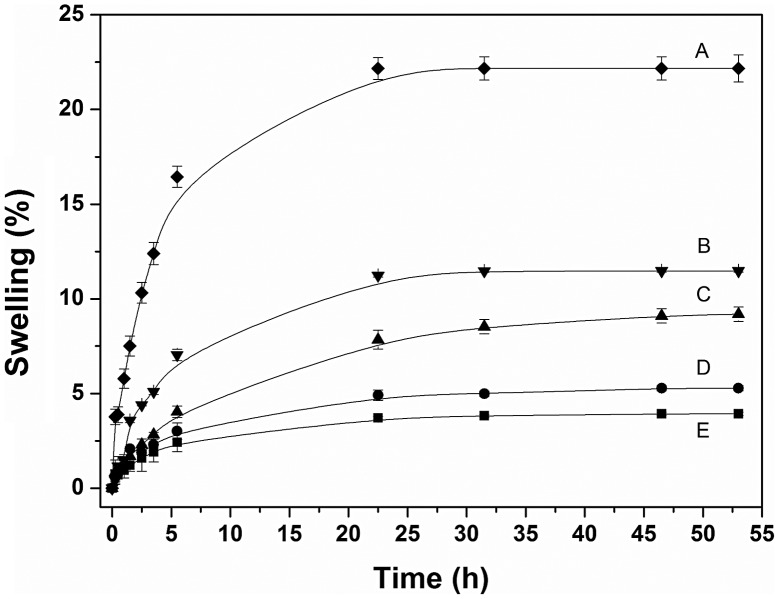
Swelling degree in water of SR-g-(NVCL-co-4VP) films with (A) 240%, (B) 80%, (C) 42%, (D) 29%, and (E) 10% grafting percentage.

When immersed in an aqueous medium SR did not absorb water. Grafting of NVCL and 4VP endowed capability to uptake water to the modified films (Figure 6). The swelling degree of grafted films increased as greater amounts of hydrophilic chains (higher grafting percentage) were grafted onto the SR surface. Water can be absorbed by the hydrophilic groups present in the grafted polymer chains via hydrogen bonding with the carbonyl groups of the lactam ring and protonation of the nitrogen atom present on the pyridine ring. The swelling process was relatively slow at 25 °C and the material reached the swelling equilibrium after 24 h.

**Figure 7.**
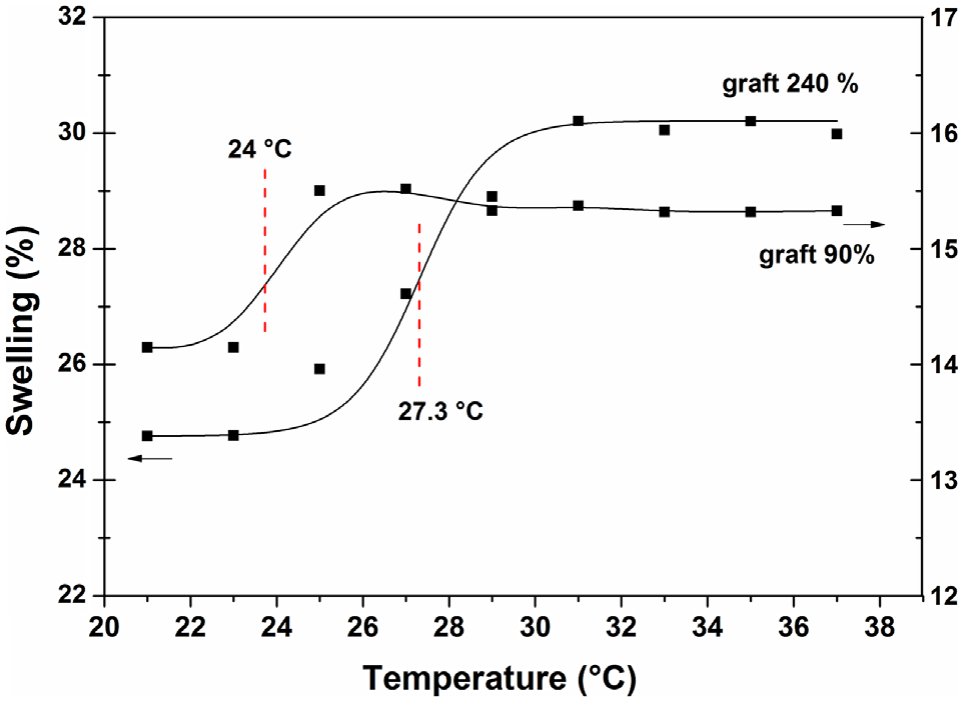
Swelling percentage in PBS (pH 7.4) as a function of temperature for SR-g-(NVCL-co-4VP) with grafting percentages of (A) 240% (*y*-axis on the left) and (B) 90% (*y*-axis on the right).

The temperature-responsive behavior of the copolymers was estimated from the temperature dependence of the swelling degree in PBS (pH 7.4) of SR-g-(NVCL-co-4VP) films. Differently from pure poly(NVCL), which exhibits a lower critical solution temperature in the 31–34 °C range,[21] SR-g-(NVCL-co-4VP) copolymers exhibited an UCST in the 24–27.3 °C range (Figure 7). Those copolymers with the highest graft percentages showed a slightly higher UCST, probably due to the effect of copolymer molecular weight on this parameter.[36] High graft percentages are not only related to more nanobrush chains, but also to longer nanobrushes. The phase transition of grafted films as a function of temperature confirmed that NVCL was grafted onto silicone. The phase transition of polyNVCL from hydrophobic to hydrophilic is due to the weakening of the associative interactions among polymer chains when the temperature is above a certain threshold, which favours the contact of individualized chains with the aqueous medium and subsequent interaction of polymer hydrophilic groups with water.[37,38]

**Figure 8.**
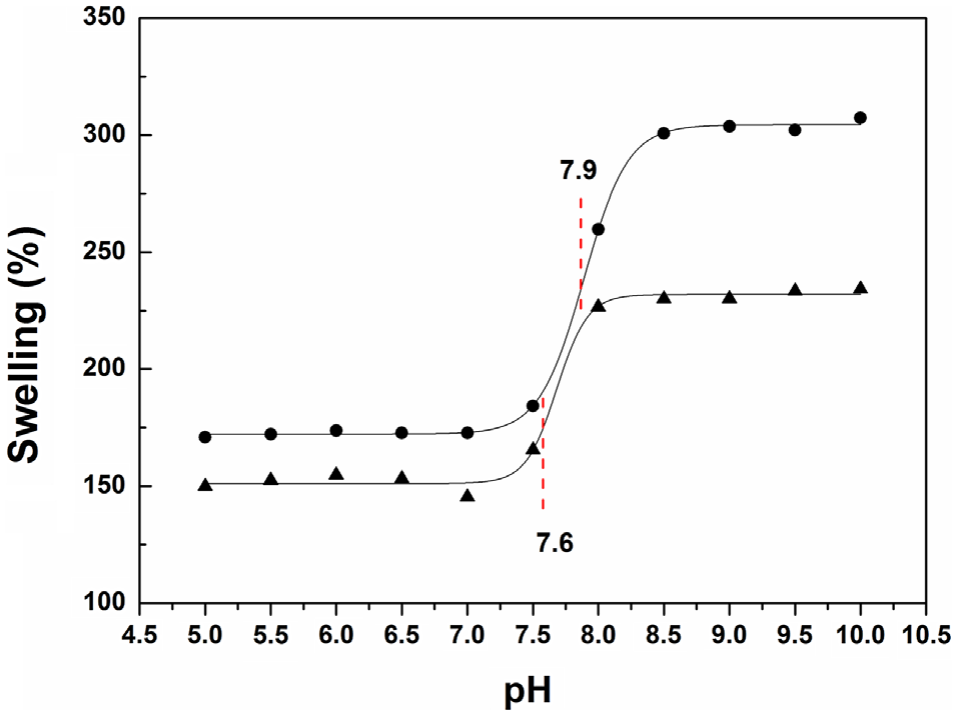
Swelling degree as a function of pH for SR-g-(NVCL-co-4VP) with grafting percentages of (A) 315% and (B) 240% at room temperature.

The swelling behavior of SR-g-(NVCL-co-4VP) as a function of pH is shown in Figure 8. The pH responsiveness was due to the 4VP moieties. A volume phase transition was recorded at a pH close to 7.8, which is slightly above the physiological value of 7.4. The degree of swelling remained almost constant up to pH 7.5, but then a sharp expansion of the network occurred. The biggest change in volume was observed for films with longer grafted chains. The critical pH may be useful for triggering a more rapid release of the drug when an increase in pH occurs, as it happens, for example, during some urinary tract infections because bacteria transform urea into ammonia and other alkaline waste products. Overall, the results obtained indicate that grafting NVCL and 4VP in one-step leads to nanobrushes that exhibit responsiveness to both temperature and pH. However, the point at which the stimuli-response took place was altered when the compounds were randomly grafted as copolymers.

### 3.4. Cytocompatibility

**Figure 9.**
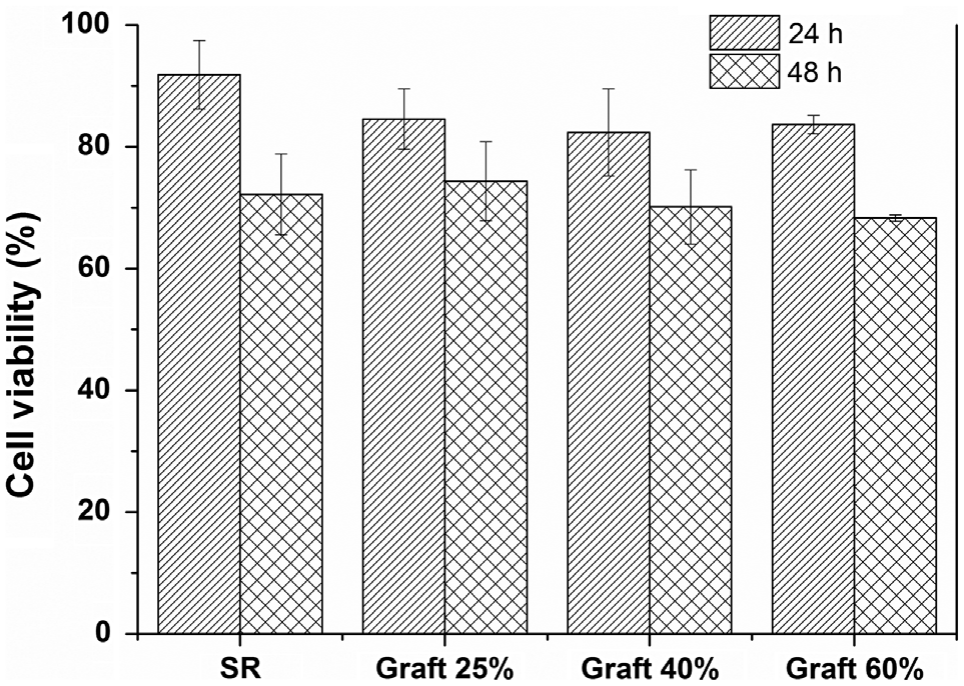
BALB 3T3 cells viability after 24 and 48 h exposition to extraction medium of copolymers prepared with different graft percentage. 100% cell viability corresponds to the negative control (i.e., cells grow under the same conditions as the cells exposed to the extraction medium of grafted polymers).

SR-g-(NVCL-co-4VP) films showed good cytocompatibility when an indirect contact method was used, which consisted in exposing Balb/3T3 cells to the extraction medium. This fibroblast cell line has been widely used to evaluate toxicity of materials due to its sensitiveness.[29] As shown in Figure 9, SR-g-(NVCL-co-4VP) films did not cause any detrimental effect on cell proliferation levels, which were similar to those recorded for pristine SR after 24 and 48 h incubation (no statistical differences for *p* < 0.05). All SR-g-(NVCL-co-4VP) films showed similar cytocompatibility disregarding the grafting percentage, with cell viability values above 80% at 24 h. Although the cell viability showed a slight decrease after 48 h, it remained above 70%.

### 3.5. Diclofenac loading and release

**Figure 10.**
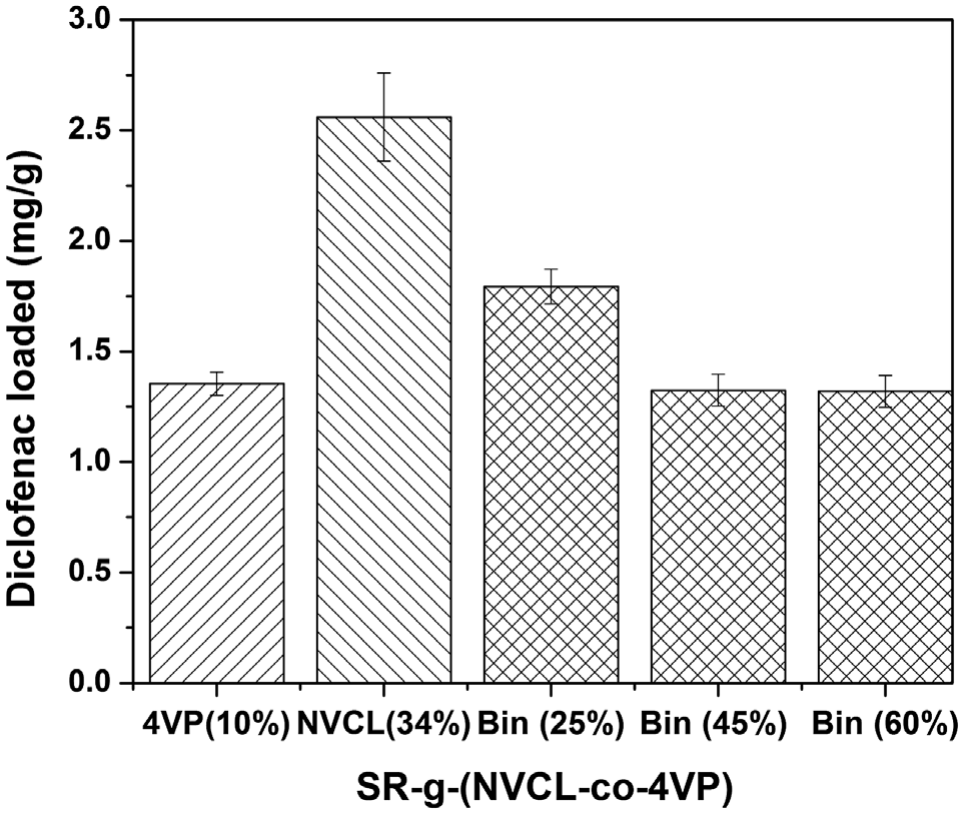
Sodium diclofenac loaded in SR-g-(NVCL-co-4VP) films with different grafting percentages. Drug loading was carried out by soaking of film pieces in aqueous solutions of diclofenac (80 mg/L) for 48 h at room temperature and protecting them from light. As controls, amounts loaded by SR-g-4VP (10% grafting) and by SR-g-NVCL (34% grafting) were also recorded.

SR did not load quantifiable amounts of diclofenac. Differently, carbonyl groups (from NVCL) and amino groups (from 4VP) made SR-g-(NVCL-co-4VP) films particularly suitable for hosting diclofenac (Figure 10). This NSAID can interact through its C–Cl bonds with the carbonyl function of NVCL, mimicking a common mechanism of interaction in the living organisms. The carboxylic acid group as well as the aromatic rings of diclofenac may establish ionic as well as hydrophobic interactions with 4VP functionalities.

Drug loading was affected by the concentration of drug in the soaking solution. Preliminary tests revealed that immersion in 40 mg/L diclofenac solution was not sufficient for saturation of the loading ability of the films, and thus a 2-fold drug concentration was used for subsequent tests. Interestingly, drug loading negatively correlated with the grafting percentage (Figure 10); SR-g-(NVCL-co-4VP) films with 25% graft loaded significantly larger amounts than 45% graft and 60% graft (*p* < 0.01). Some control experiments were carried out with SR films that were grafted with 4VP or NVCL in separate in order to confirm that both components of the grafted layer in SR-g-(NVCL-co-4VP) films were able to host diclofenac. SR-g-NVCL 34% graft showed an statistically greater loading (*p* < 0.01) than the others films. The decrease in diclofenac loading as NVCL-co-4VP grafting increased suggests that denser and longer nanobrushes cause steric hindrance to drug penetration in the grafted layer. Nevertheless, even for the SR-g-(NVCL-co-4VP) film with the highest grafting percentage tested, the amount of diclofenac loaded was above 1 mg/g, which may be suitable for obtaining therapeutic effects.[22,31,39]

**Figure 11.**
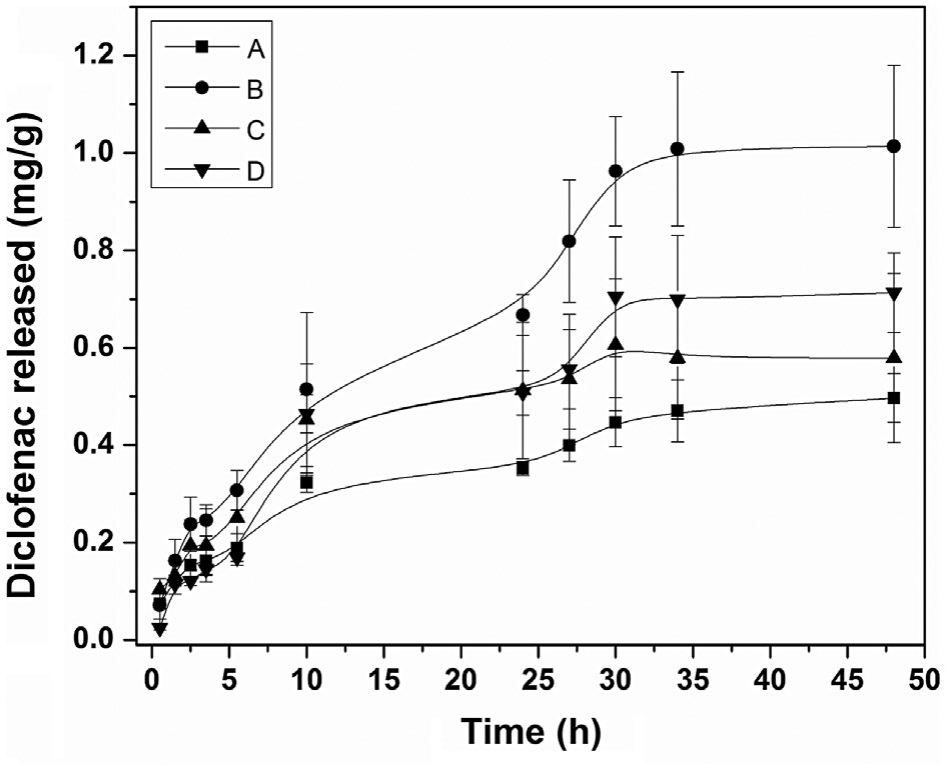
Diclofenac release profiles in 0.9% NaCl medium at 37 °C from drug-loaded SR-g-4VP 12% graft (A), SR-g-NVCL 34% graft (B), SR-g-(NVCL-co-4VP) 45% graft (C), and SR-g-(NVCL-co-4VP) 25% graft (D).

Drug release was carried out at 37 °C in serum saline medium under sink conditions (Figure 11). SR-g-(NVCL-co-4VP) films sustained drug release for at least two days. Therefore, although the conditions used for the release tests (mimicking physiological ones) may trigger greater swelling of the grafted films, the affinity of both NVCL and 4VP for the drug still controls drug release for prolonged time. Once a medical device is implanted or inserted in the body, first hours are critical for foreign body reactions and bacteria adhesion. Thus, the performance of SR-g-(NVCL-co-4VP) films regulating diclofenac release in those first hours/days may prevent undesirable events.

### 3.6. Bacterial adhesion assay

One should keep in mind that grafting of swellable nanobrushes to endow medical devices with drug-eluting performance may have the negative impact of that porous network facilitates bacteria adhesion and growth onto the device. To gain an insight into the feasibility of diclofenac-loaded SR-g-(NVCL-co-4VP) films to diminish the risk of bacteria adhesion, pristine and modified films were challenged against adhesion of an *E. coli* strain. The experiments were carried out incubating the films in a high concentration *E. coli* medium for 3 h. Pristine SR did not cause any decrease in bacterial growth. Compared to non-loaded modified films, diclofenac-loaded counterparts were less prone to bacteria adhesion (Table 1; *p* < 0.01). Drug-loaded SR-g-(NVCL-co-4VP) films with 60% graft caused more than 1 log decrease in bacteria adhesion (namely, 91% less adhered bacteria).

**Table 1.** CFU of *E. coli* to measure its microbial adhesion onto modified films.

Film	CFU without drug	CFU with drug
SR-g-(NVCL-co-4VP) (24%)	2.7 × 10^9^	7.7 × 10^8^
SR-g-(NVCL-co-4VP) (60%)	7.8 × 10^9^	6.7 × 10^8^

## 4. Conclusions

NVCL and 4VP can be simultaneously grafted onto SR films in one-step using an irradiation grafting method where gamma rays serve as the reaction initiator. Tuning absorbed dose and monomer concentration a variety of grafting percentages can be achieved. SR-g-(NVCL-co-4VP) films are more hydrophilic than SR films and show temperature and pH responsiveness. The new graft copolymer exhibits moderate thermal stability, with a decomposition temperature between 382 and 403 °C (10 wt% loss). Moreover, SR-g-(NVCL-co-4VP) films can host relevant amounts of diclofenac and release it in a controlled manner, diminishing bacterial adhesion.

## Disclosure statement

No potential conflict of interest was reported by the authors.

## Funding

This work was supported by DGAPA-UNAM [grant number IN200714] (México); MICINN [grant number SAF2014-52632-R] (Spain); FEDER; and the Ibero-American Program for Science, Technology and Development of CYTED (‘Red iberoamericana de nuevos materiales para el diseño de sistemas avanzados de liberación de fármacos en enfermedades de alto impacto socioeconómico’ RIMADEL).
